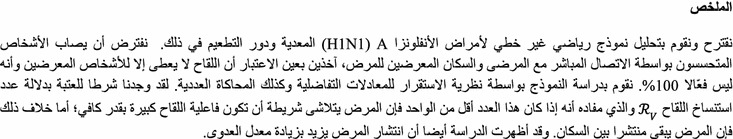# Analysis of an influenza A (H1N1) epidemic model with vaccination

**DOI:** 10.1007/s40065-012-0013-6

**Published:** 2012-04-19

**Authors:** Xueyong Zhou, Zhen Guo

**Affiliations:** 1 School of Mathematical Sciences, Nanjing Normal University, Nanjing, 210046 Jiangsu People’s Republic of China; 2 College of Mathematics and Information Science, Xinyang Normal University, Xinyang, 464000 Henan People’s Republic of China; 3 School of Computer and Information Technology, Xinyang Normal University, Xinyang, 464000 Henan People’s Republic of China

**Keywords:** 92B05, 34D05

## Abstract

A nonlinear mathematical model for the spread of influenza A (H1N1) infectious diseases including the role of vaccination is proposed and analyzed. It is assumed that the susceptibles become infected by direct contact with infectives and exposed population. We take under consideration that only a susceptible person can be vaccinated and that the vaccine is not 100% efficient. The model is analyzed using stability theory of differential equations and numerical simulation. We have found a threshold condition, in terms of vaccination reproduction number ${\mathcal{R}_V}$ which is, if less than one, the disease dies out provided the vaccine efficacy is high enough, and otherwise the infection is maintained in the population. It is also shown that the spread of an infectious disease increases as the infective rate increases.

**Figure:**

**Mathematics Subject Classification (2010)** 92B05 · 34D05

**الملخص**

نقترح ونقوم بتحليل نموذج رياضي غير خطي لأمراض الأنفلونزا A (H1N1) المعدية ودور التطعيم في ذلك. نفترض أن يصاب الأشخاص المتحسسون بواسطة الاتصال المباشر مع المرضى والسكان المعرضين للمرض، آخذين بعين الاعتبار أن اللقاح لا يعطى إلا للأشخاص المعرضين وأنه ليس فعّالا 100%. نقوم بدراسة النموذج بواسطة نظرية الاستقرار للمعادلات التفاضلية وكذلك المحاكاة العددية. لقد وجدنا شرطا للعتبة بدلالة عدد استنساخ اللقاح $\mathcal{R}_V$ والذي مفاده أنه إذا كان هذا العدد أقل من الواحد فإن المرض يتلاشى شريطة أن تكون فاعلية اللقاح كبيرة بقدر كافي؛ أما خلاف ذلك فإن المرض يبقى منتشرا بين السكان. وقد أظهرت الدراسة أيضا أن انتشار المرض يزيد بزيادة معدل العدوى.

## 1 Introduction

Influenza, also called the flu, is an illness caused by a virus that affects mainly the nose, throat, bronchi and, occasionally, lungs. Infection usually lasts for about a week, and is characterized by fever, myalgia, headache, pharyngitis, cough and prostration. Influenza is highly contagious and is easily transmitted through contact

X. Zhou (✉)
School of Mathematical Sciences, Nanjing Normal University,
Nanjing 210046, Jiangsu, People's Republic of China
E-mail: xueyongzhou@126.com

X. Zhou
College of Mathematics and Information Science, Xinyang Normal University,
Xinyang 464000, Henan, People's Republic of China

Z. Guo
School of Computer and Information Technology, Xinyang Normal University,
Xinyang 464000, Henan, People's Republic of China with droplets from the nose and throat of an infected person who is coughing and sneezing. The disease infects the nose, throat or lungs. It often breaks out as an epidemic which quickly spreads from town to town and country to country. Typically, an area can have epidemic conditions for a period of 4–6 weeks before it eases off. As it is so easily spread, influenza can make many people ill in a short period of time. Onset of symptoms range from 18 to 72 h.

Major pandemics have occurred in 1889, 1918–1919, 1957 and 1968 and another major pandemic may well occur in the near future. The 1918–1919 pandemic of Spanish flu was one of the most devastating epidemics of an infectious disease to have affected mankind [11]. This pandemic was directly responsible for over 20 million deaths, more than perished in conflict in the preceding Great War, and mainly affected previously healthy young adults [8,13].

Human cases of swine influenza A (H1N1) virus infection have been identified recently in several countries. Cases of novel H1N1 influenza were first identified in mid-April 2009 in California and soon thereafter in Texas and Mexico [14]. The earliest recognized case occurred in Mexico with illness onset on March 17, 2009. Since that time, the virus has spread across the globe, and on June 11, 2009, the World Health Organization (WHO) declared the onset of an influenza pandemic. This H1N1 pandemic is spreading far and wide and moving fast. The number of human cases of pandemic H1N1 is still increasing substantially in many countries, even in countries that have already been affected for some time. As of 4 October 2009, worldwide there have been more than 375,000 laboratory confirmed cases of pandemic influenza H1N1 and over 4,500 deaths reported to WHO [20].

Since the newly infected cases become more than the imported cases, the control of the spread of H1N1 becomes urgent. Most of the countries have activated their pandemic preparedness plans, such as heightened surveillance, early detection and treatment of cases, and infection control in all health facilities.

Vaccination is the best protection against contracting the H1N1 (Swine) flu [3]. On 13 July 2009, the WHO recommended that all countries immunize their health care workers (HCWs) as a first priority in order to protect the vital health infrastructure and patients [21].

In order to consider the effect of influenza A (H1N1), in this paper, we will consider an influenza A (H1N1) model with vaccination.

Let $S(t)$ be the number of population members who are susceptible to an infection at time $t$, $V(t)$ be the number of members who are vaccinated at time $t$, $E(t)$ be the number of members who are latened at time $t$, $I(t)$ be the number of members who are infective at time $t$ and $R(t)$ be the number of members who are recovered at time $t$. Hence, the total population at the time $t$, $N(t)$, is now given by $N(t) = S(t)+V(t)+E(t)+I(t)+R(t)$.

Our model is based on the following assumptions.

1. The population of the susceptible population is generated by the birth or immigration at a rate $A$. This population is reduced by infection, vaccination and natural death. The infection can be acquired following effective contact with infected population and exposed population: $\beta(I+\eta E)$. The parameter $\beta$ is the rate at which susceptible individuals become infected by those who are infectious. Further, the modification parameter $0 \le \eta \le 1$ accounts for the assumed increase in the relative infectiousness of population in the $I$ classes in comparison to those in the corresponding exposed classes $E$. Susceptible individuals are vaccinated at a rate $\phi$. And the population is reduced by natural death at a rate $\mu$. Thus,
$$\frac{dS}{dt} = A - \beta(I+\eta E)S - (\phi+\mu)S.$$
2. The population of vaccinated individuals is increased by vaccination of susceptibles at a rate $\phi$. Since the vaccination is assumed to be imperfect, vaccinated individuals can acquire breakthrough infection at a reduced rate $(1-\sigma)\beta(I+\eta E)$. And the vaccinated class is diminished by this infection (moving to class $E$) and by natural death at a rate $\mu$. The vaccine has the effect of reducing infection by a factor of $\sigma$ $(0 < \sigma < 1)$, so that $\sigma = 1$ means that the vaccine in completely effective in preventing infection, while $\sigma = 0$ means that the vaccine is utterly ineffective, thus in reality $0 < \sigma < 1$. Hence,
$$\frac{dV}{dt} = \phi S - (1-\sigma)\beta(I+\eta E)V - \mu V.$$
3. The exposed population is increased due to the infection of infected and exposed population. This population is reduced due to development of clinical symptoms (at a rate $k_1$) and natural death (at the rate $\mu$). Thus,
$$\frac{dE}{dt} = \beta(I+\eta E)S + (1-\sigma)\beta(I+\eta E)V - k_1 E - \mu E.$$

**Fig. 1** Transfer diagram of the influenza A (H1N1) epidemic model

**Table 1** Description of variables and parameters of the vaccination model (1.1)

| Variable | Description | | |
|---|---|---|---|
| $S(t)$ | Susceptible individuals | | |
| $V(t)$ | Vaccinated individuals | | |
| $E(t)$ | Exposed individuals | | |
| $I(t)$ | Infected individuals | | |
| $R(t)$ | Recovered individuals | | |
| Parameters | Description | Values | Reference |
| $A$ | Birth or immigrant rate | 15/day | Assumed |
| $\beta$ | Infection rate | variable | |
| $\eta$ | Modification parameter | 0.3 | Assumed |
| $\phi$ | Vaccination rate of susceptible individuals | 0.01 | [1] |
| $\mu$ | Natural death rate | $5.48\times10^{-5}$/day | [12] |
| $\sigma$ | Factor by which the vaccine reduces infection | 0.15/day | Assumed |
| $d$ | Disease-induced death rate | 0.001/day | [16] |
| $k_1$ | Transfer rates between the exposed and the infectious | 0.2/day | [17] |
| $\delta$ | Rate of recovery from the disease | 0.14/day | [10] |

4. The infectious population is generated following the development of disease symptoms by exposed individuals (at the rate $k_1$). It is reduced by natural death (at the rate $\mu$), disease-induced death (at a rate $d$), or recovery from the disease (at a rate $\delta$), so that

$$\frac{dI}{dt} = k_1 E - dI - \delta I - \mu I.$$

5. The recovered population is generated following the recovery of infectious population (at the rate $\delta$) and reduced due to natural death (at the rate $\mu$). Hence,

$$\frac{dR}{dt} = \delta I - \mu R.$$

Thus, the model for the transmission dynamics of influenza A (H1N1) in a population is given by the following system of non-linear differential equations:

$$\begin{cases} \frac{dS}{dt} = A - \beta(I+\eta E)S - (\phi+\mu)S, \\ \frac{dV}{dt} = \phi S - (1-\sigma)\beta(I+\eta E)V - \mu V, \\ \frac{dE}{dt} = \beta(I+\eta E)S + (1-\sigma)\beta(I+\eta E)V - k_1 E - \mu E, \\ \frac{dI}{dt} = k_1 E - dI - \delta I - \mu I, \\ \frac{dR}{dt} = \delta I - \mu R. \end{cases} \tag{1.1}$$

The transfer diagram for these processes is shown in Fig. 1. And the associated model variable and parameters are described in Table 1. All parameters of the model (1.1) are assumed to be nonnegative.

Note that the recovered population $R$ does not appear in the first four equations. Thus, we can consider the following sub-system of system (1.1):

$$\begin{cases} \frac{\mathrm{d}S}{\mathrm{d}t} = A - \beta(I + \eta E)S - (\phi + \mu)S, \\ \frac{\mathrm{d}V}{\mathrm{d}t} = \phi S - (1 - \sigma)\beta(I + \eta E)V - \mu V, \\ \frac{\mathrm{d}E}{\mathrm{d}t} = \beta(I + \eta E)S + (1 - \sigma)\beta(I + \eta E)V - k_1 E - \mu E, \\ \frac{\mathrm{d}I}{\mathrm{d}t} = k_1 E - \mathrm{d}I - \delta I - \mu I. \end{cases} \tag{1.2}$$

The initial conditions of system (1.1) are presented in the following:

$$S(0) \geq 0, \quad V(0) \geq 0, \quad E(0) \geq 0, \quad I(0) \geq 0, \quad R(0) \geq 0. \tag{1.3}$$

## 2 Basic properties of the system (1.2)

### 2.1 Positivity and boundedness of solutions

Let $\lambda(t) = \beta(I(t) + \eta E(t))$.

**Theorem 2.1** *The solutions* $(S, V, E, I)$ *of the system (1.2) are non-negative with non-negative initial conditions for all* $t > 0$. *Furthermore,*

$$\limsup_{t\to\infty} N(t) \leq \frac{A}{\mu}.$$

*Proof* Let $T = \sup\{t > 0 : S, V, E, I > 0\}$. Thus, $T > 0$. It follows from the first equation of the differential equation system (1.2) that

$$\frac{\mathrm{d}S}{\mathrm{d}t} = A - [\lambda(t) + \phi + \mu]S,$$

which is equivalent to

$$\frac{\mathrm{d}}{\mathrm{d}t}\left[S(t)\exp\left\{\int_0^t \lambda(u)\mathrm{d}u + (\phi + \mu)t\right\}\right] = A\exp\left\{\int_0^t \lambda(u)\mathrm{d}u + (\phi + \mu)t\right\}.$$

Thus,

$$S(T)\exp\left\{\int_0^T \lambda(u)\mathrm{d}u + (\phi + \mu)T\right\} - S(0) = \int_0^T A\exp\left\{\int_0^x \lambda(v)\mathrm{d}v + (\phi + \mu)x\right\}\mathrm{d}x,$$

so that

$$\begin{aligned} S(T) &= S(0)\exp\left\{-\int_0^T \lambda(u)\mathrm{d}u + (\phi + \mu)T\right\} + \exp\left\{-\int_0^T \lambda(u)\mathrm{d}u + (\phi + \mu)T\right\} \\ &\quad \times \int_0^T A\exp\left\{\int_0^x \lambda(v)\mathrm{d}v + (\phi + \mu)x\right\}\mathrm{d}x > 0. \end{aligned}$$

Similarly, it can be shown that $V > 0$, $E > 0$ and $I > 0$ for all $t > 0$. Thus, all solutions of the model, with non-negative initial data, remain non-negative for all $t > 0$. □

## 2.2 Invariant region and attractiveness

Adding all the equations of the system (1.2) gives that

$$\frac{\mathrm{d}N(t)}{\mathrm{d}t} = A - \mu N - \mathrm{d}I - \delta I. \tag{2.1}$$

Noting that $0 < I(t) \leq N(t)$, it follows from (2.1) that

$$A - (\mu + \delta + 2d)N(t) \leq \frac{\mathrm{d}N(t)}{\mathrm{d}t} \leq A - \mu N(t).$$

Thus,

$$\frac{A}{\mu + 2d + \delta} \leq \liminf_{t\to\infty} N(t) \leq \limsup_{t\to\infty} N(t) \leq \frac{A}{\mu},$$

so that

$$\limsup_{t\to\infty} N(t) = \frac{A}{\mu},$$

as required.

From the first equation of (1.2), it follows that

$$0 < \limsup_{t\to\infty} S(t) \leq \frac{A}{\mu + \phi} \tag{2.2}$$

and then from the second equation of (1.2),

$$0 < \limsup_{t\to\infty} V(t) \leq \frac{A\phi}{\mu(\mu + \phi)}. \tag{2.3}$$

It follows from (2.1) that if $N > \frac{A}{\mu}$, then $\frac{\mathrm{d}N}{\mathrm{d}t} < 0$.

The system (1.2) will be analyzed in biologically feasible region as follows. Consider the feasible region

$$\Omega = \left\{(S, V, E, I) \in \mathcal{R}_4^+ : S + V + E + I \leq \frac{A}{\mu}, S \leq \frac{A}{\mu + \phi}, V \leq \frac{A\phi}{\mu(\mu + \phi)}\right\}.$$

From the above, we can establish the following theorem.

**Theorem 2.2** *The region $\Omega$ is positively invariant for the system* (1.2) *with initial conditions in $\mathcal{R}_4^+$.*

## 3 Stability of disease-free equilibrium

This metric is useful because it helps determine whether or not an infectious disease will spread through a population. In this section, we will calculate the basic reproduction number of the system (1.2).

It is easy to see that the system (1.2) always has a disease-free equilibrium (the absence of infection, i.e., $E = I = 0$), $P_0(S_0, V_0, 0, 0)$, where $S_0 = \frac{A}{\mu+\phi}$, $V_0 = \frac{A\phi}{\mu(\mu+\phi)}$.

Let $x = (E, I, S, V)^\top$. Then the system (1.2) can be written as

$$\frac{\mathrm{d}x}{\mathrm{d}t} = \mathcal{F}(x) - \mathcal{V}(x),$$

where

$$\mathcal{F}(x) = \begin{pmatrix} \beta(I + \eta E)S + (1 - \sigma)\beta(I + \eta E)V \\ 0 \\ 0 \\ 0 \end{pmatrix},$$

$$\mathcal{V}(x) = \begin{pmatrix} k_1 E + \mu E \\ -k_1 E + \mathrm{d}I + \delta I + \mu I \\ -A + \beta(I + \eta E)S + (\phi + \mu)S \\ -\phi S + (1-\sigma)\beta(I + \eta E)V + \mu V \end{pmatrix}.$$

We can get

$$\mathbf{F} = \begin{pmatrix} \beta[S_0 + (1-\sigma)V_0] & \eta\beta[S_0 + (1-\sigma)V_0] \\ 0 & 0 \end{pmatrix}, \quad \mathbf{V} = \begin{pmatrix} k_1 + \mu & 0 \\ -k_1 & \mu + d + \delta \end{pmatrix},$$

giving

$$\mathbf{V}^{-1} = \begin{pmatrix} \frac{1}{\mu+k_1} & 0 \\ \frac{k_1}{(\mu+k_1)(\mu+d)} & \frac{1}{\mu+d+\delta} \end{pmatrix}.$$

$\mathbf{FV}^{-1}$ is the next generation matrix for model (1.2). It then follows that the spectral radius of matrix $\mathbf{FV}^{-1}$ is $\rho(\mathbf{FV}^{-1}) = \frac{\beta[S_0+(1-\sigma)V_0]}{\mu+k_1} + \frac{k_1\eta\beta[S_0+(1-\sigma)V_0]}{(\mu+k_1)(\mu+d+\delta)}$. According to Theorem 2 in [19], the vaccination reproduction number of model (1.2) is

$$\begin{aligned} \mathcal{R}_V &= \frac{\beta[S_0 + (1-\sigma)V_0]}{\mu + k_1} + \frac{k_1\eta\beta[S_0 + (1-\sigma)V_0]}{(\mu + k_1)(\mu + d + \delta)} \\ &= \frac{\beta A(\mu + \phi - \sigma\phi)(\mu + d + \delta + k_1\eta)}{\mu(\mu + \phi)(\mu + k_1)(\mu + d + \delta)}. \end{aligned} \tag{3.1}$$

The vaccination reproduction number $\mathcal{R}_V$ can be interpreted as the average total number of new infections caused by infected and exposed individual, introduced into a susceptible population in which some individuals have been vaccinated.

It is easy to show that $\mathcal{R}_V > 1$ (since all the parameters of the model are non-negative). Thus, by Theorem 2 in [19], the following result is established.

**Theorem 3.1** *The disease-free disease equilibrium, $P_0$, of the model (1.2) is locally asymptotically stable if $\mathcal{R}_V < 1$ and unstable if $\mathcal{R}_V > 1$.*

From Sect. 2, we can see $0 < \limsup_{t\to\infty} S(t) \le S_0$ and $0 < \limsup_{t\to\infty} V(t) \le V_0$. Hence, from the last two equations of system (1.2), we can obtain

$$\begin{cases} \frac{\mathrm{d}E}{\mathrm{d}t} \le \frac{\beta A}{\mu+\phi}(I + \eta E) + \frac{(1-\sigma)\beta A}{\mu(\mu+\phi)}(I + \eta E) - k_1 E - \mu E, \\ \frac{\mathrm{d}I}{\mathrm{d}t} = k_1 E - \mathrm{d}I - \delta I - \mu I. \end{cases} \tag{3.2}$$

Using a standard comparison argument [9], we only need to show that every solution of the system

$$\begin{cases} \frac{\mathrm{d}X}{\mathrm{d}t} = \frac{\beta A}{\mu+\phi}(X + \eta Y) + \frac{(1-\sigma)\beta A}{\mu(\mu+\phi)}(X + \eta Y) - k_1 X - \mu X, \\ \frac{\mathrm{d}Y}{\mathrm{d}t} = k_1 X - \mathrm{d}Y - \delta Y - \mu Y \end{cases} \tag{3.3}$$

converges to zero as $t \to \infty$.

It is easy to see that every solution of the system (3.3) converges to zero as $t \to \infty$ when $\mathcal{R}_V < 1$. Hence, $I \le \epsilon_1$, $E \le \epsilon_2$ for large $t$ and sufficiently small $\epsilon_1 > 0$ and $\epsilon_2 > 0$. Thus, the first equation of (1.2) can be expressed as

$$\frac{\mathrm{d}S}{\mathrm{d}t} \ge A - \beta(\epsilon_1 + \eta\epsilon_2)S - (\phi + \mu)S. \tag{3.4}$$

Then,

$$\liminf_{t\to\infty} S(t) \ge \frac{A}{\beta(\epsilon_1 + \eta\epsilon_2) + \phi + \mu}. \tag{3.5}$$

From the second equation of (1.2), we can get

$$\frac{\mathrm{d}V}{\mathrm{d}t} = \frac{\phi A}{\beta(\epsilon_1 + \eta\epsilon_2) + \phi + \mu} - (1-\sigma)\beta(\epsilon_1 + \eta\epsilon_2)V - \mu V.$$

Hence,

$$\liminf_{t\to\infty} V(t) \geq \frac{\phi A}{[\beta(\epsilon_1 + \eta\epsilon_2) + \phi + \mu][(1-\sigma)\beta(\epsilon_1 + \eta\epsilon_2)V + \mu]}.$$

Setting $\epsilon_1 \to 0$ and $\epsilon_2 \to 0$ gives

$$\liminf_{t\to\infty} S(t) \geq \frac{A}{\phi + \mu}, \quad \liminf_{t\to\infty} V(t) \geq \frac{A\phi}{\mu(\phi + \mu)}.$$

Combining (2.2) and (2.3) shows that if $\mathcal{R}_V < 1$,

$$\lim_{t\to\infty} S(t) = \frac{A}{\phi + \mu}, \quad \lim_{t\to\infty} V(t) = \frac{A\phi}{\mu(\phi + \mu)}.$$

Thus, $P_0$ is globally asymptotically stable in $\Omega$ whenever $\mathcal{R}_V < 1$.

## 4 Endemic equilibrium

To establish the existence of endemic equilibria of the model (1.2), the following steps are considered. Let $P^* = (S^*, V^*, E^*, I^*)$ represent any arbitrary endemic equilibrium of the vaccination model (1.2). Further, consider

$$\lambda^* = \beta(I^* + \eta E^*). \tag{4.1}$$

It follows, by solving the equations in (1.2) at steady state, that the non-zero equilibria of the model satisfy the following quadratic equation (in terms of $\lambda^*$)

$$E^* = \frac{[(1-\sigma)\lambda^* + \mu + \phi\sigma]\lambda^* A}{(\mu + k_1)(\mu + \sigma\lambda^*)(\lambda^* + \mu + \phi)},$$

$$I^* = \frac{k_1[(1-\sigma)\lambda^* + \mu + \phi\sigma]\lambda^* A}{(\mu + k_1)(\mu + \sigma\lambda^*)(\lambda^* + \mu + \phi)(\mu + d + \delta)}$$

and

$$a_1\lambda^{*2} + a_2\lambda^* + a_3 = 0, \tag{4.2}$$

where

$$a_1 = (1-\sigma)(\mu + k_1)(\mu + d + \delta),$$
$$a_2 = (\mu + k_1)(\mu + d + \delta)(\mu + \phi)[\mu + (1-\sigma)(\mu + \phi)] - (1-\sigma)\beta A(\mu + d + \delta + k_1\eta),$$
$$a_3 = \mu(\mu + k_1)(\mu + d + \delta)(\mu + \phi)(1 - \mathcal{R}_V).$$

Now an endemic equilibrium of (1.2) can be calculated from a positive real solution of Eq. (4.2). It is easy to see that $a_1 > 0$ and $a_3 > 0$ precisely when $\mathcal{R}_V < 1$. And $a_2^2 - 4a_1a_3 > 0$ when $a_3 < 0$. One can easily deduce that there is precisely one endemic equilibrium when $\mathcal{R}_V > 1$, since there are two real roots and the product of those two roots is negative. On the other hand, we can see that $a_3 < 0$ precisely when $\mathcal{R}_V > 1$. Note that there are exactly two changes in the sign of coefficients of Eq. (4.2) if coefficient $a_2 < 0$ and none when $a_2 > 0$. By Descartes' rule of signs [2], one can conclude that the maximum number of endemic equilibria is two when $\mathcal{R}_V < 1$ and $a_2 < 0$, and that there is no endemic equilibrium when $\mathcal{R}_V < 1$ and $a_2 > 0$. However, it is shown that it is always the case that the system does not have any endemic equilibrium when $\mathcal{R}_V < 1$.

**Theorem 4.1** *For system* (1.2)*, there is no endemic equilibrium when* $\mathcal{R}_V < 1$.

*Proof* First, we assume that $\mathcal{R}_V < 1$ and $a_2 < 0$:

$$\mathcal{R}_V < 1 \Leftrightarrow \beta A < \frac{\mu(\mu+k_1)(\mu+d+\delta)(\mu+\phi)}{(\mu+\sigma\phi)(\mu+d+\delta+\eta k_1)},$$
$$a_2 < 0 \Leftrightarrow \beta A > \frac{(\mu+k_1)(\mu+d+\delta)[\mu+\sigma(\mu+\phi)]}{\sigma(\mu+d+\delta+\eta k_1)}.$$

Combining these two conditions, one can get the following relation:

$$\frac{\mu+\sigma(\mu+\phi)}{\sigma} < \frac{\sigma(\mu+\phi)}{\mu+\sigma\phi}.$$

After some calculations we can get

$$\mu^2 + \mu\sigma\phi + \sigma^2\phi(\mu+\phi) < 0,$$

which is a contradiction for all nonzero parameters. Therefore we can rule out the case where $\mathcal{R}_V < 1$ and $a_2 < 0$. Note that $\mathcal{R}_V < 1$ corresponds to $a_3 > 0$. One can deduce that when $\mathcal{R}_V < 1$, $a_2$ is always positive for nonzero parameters, thus we cannot have any endemic equilibrium in this case by Descartes' rule of signs. □

## 5 Persistence

In this section we will present the persistence of system (1.2). Firstly, we recall some definitions in order to introduce the abstract theorem. Assume $Y$ is a locally compact metric space with metric d and let $F$ be a closed subset of $Y$ with boundary $\partial F$ and interior int$F$. Let $\pi$ be a semi-dynamical system defined on $F$.

We say that $\pi$ is persistent if for all $u \in \text{int}F$, $\liminf_{t\to+\infty} \mathrm{d}(\pi(u,t), \partial F) > 0$ and that $\pi$ is uniformly persistent if there is $\epsilon > 0$ such that for all $u \in \text{int}F$, $\liminf_{t\to+\infty} \mathrm{d}(\pi(u,t), \partial F) > \epsilon$.

In [4], Fonda gives a result about persistence in terms of repellers. A subset $\Sigma$ of $F$ is said to be a uniform repeller if there is a $\epsilon > 0$ such that, for each $u \in F\backslash\Sigma$, $\liminf_{t\to+\infty} \mathrm{d}(\pi(u,t), \Sigma) > \epsilon$. A semiflow on a closed subset $F$ of a locally compact metric space is uniformly persistent if the boundary of $F$ is repelling. The result of Fonda is the following.

**Lemma 5.1** [4] *Let $\Sigma$ be a compact subset of $Y$ such that $Y \setminus \Sigma$ is positively invariant. A necessary and sufficient condition for $\Sigma$ to be a uniform repeller is that there exists a neighborhood $U$ of $\Sigma$ and a continuous function $P : Y \to \mathcal{R}^+$ satisfying*

*(i) $P(u) = 0$ if and only if $u \in \Sigma$.*
*(ii) For all $u \in U \setminus \Sigma$ there is a $T_u$ such that $P(\pi(u, T_u)) > P(u)$.*

*For any $u_0 = (S(0), V(0), E(0), I(0)) \in \Omega$ there is a unique solution $\pi(u_0, t) = (S, V, E, I)(t; u_0)$ of the system (1.2), which is defined in $\mathcal{R}^+$ and satisfies $\pi(u_0, 0) = (S(0), V(0), E(0), I(0))$. Since $\Omega$ is a positively invariant set of the system (1.2), then $\pi(u_0, t) \in \Omega$ for $t \in \mathcal{R}^+$ and is a semi-dynamical system in $\Omega$.*

We are now in a position to prove that $\Sigma = \{(S, V, E, I) \in \Sigma : I = 0\}$ is a uniform repeller, which implies that the semi-dynamic system $\pi$ is uniformly persistent.

**Theorem 5.1** *If $\mathcal{R}_v > 1$, then the set $\Sigma$ is a uniform repeller and hence $\pi$ is uniformly persistent in $\Omega$.*

*Proof* It is easy to show that $I(t) > 0$ for $t > 0$ if $I(0) > 0$, so $\Omega \setminus \Sigma$ is positively invariant. Again the set $\Sigma$ is a compact subset of $\Omega$. Let $P : \Omega \to \mathcal{R}^+$ be defined by $P(S, V, E, I) = I$ and $U = \{(S, V, E, I) \in \Omega : P(S, V, E, I) < \xi\}$, where $\xi > 0$ is small enough.

Assume that there is $\bar{u} \in U$ $(\bar{u} = (\bar{S}, \bar{V}, \bar{E}, \bar{I}))$ such that for each $t > 0$ we have $P(\pi(\bar{u}, t)) < P(\bar{u}) < \xi$, which implies that $I(t; \bar{u}) < \xi$ for $t > 0$. From the first two equations of the system (1.2) we have

$$\begin{cases} \frac{\mathrm{d}S(t)}{\mathrm{d}t} \geq A - (\phi+\mu)S, \\ \frac{\mathrm{d}V(t)}{\mathrm{d}t} \geq \phi S - \mu V, \end{cases}$$

then $\liminf_{t\to\infty} S(t, \bar{u}) \geq \frac{A}{\phi+\mu}$, $\liminf_{t\to\infty} V(t, \bar{u}) \geq \frac{\phi A}{\mu(\mu+\phi)}$. So there is a sufficiently large $T > 0$ such that $S(t, \bar{u}) \geq \frac{A}{\phi+\mu} - \epsilon$ and $V(t, \bar{u}) \geq \frac{\phi A}{\mu(\mu+\phi)} - \epsilon$ for $t > T$, where $\epsilon > 0$ is a sufficiently small constant.

Define the auxiliary function

$$L(t) = \frac{k_1(1-\xi^*)}{k_1+\mu}E(t) + I(t),$$

where $\xi^*(0 < \xi^* < 1)$ is a sufficiently small constant so that $\frac{k_1(1-\xi^*)}{k_1+\mu}(\beta(\frac{A}{\phi+\mu}-\epsilon)+(1-\sigma)\beta(\frac{\phi A}{\mu(\mu+\phi)}-\epsilon)) - (d+\mu+\delta) > 0$, since $\mathcal{R}_V > 1$. Calculating directly gives the derivative of $L(t)$ along $\pi(\bar{u}, t)$ as follows:

$$\begin{aligned}
\frac{\mathrm{d}L(t)}{\mathrm{d}t} &= \frac{k_1(1-\xi^*)}{k_1+\mu}[\beta(I+\eta E)S(t) + (1-\sigma)\beta(I+\eta E)V(t) - (k_1+\mu)E(t)] \\
&\quad + k_1E(t) - (d+\mu+\delta)I(t) \\
&= \frac{k_1(1-\xi^*)}{k_1+\mu}[\beta(I+\eta E)S(t) + (1-\sigma)\beta(I+\eta E)V(t)] + k_1\xi^*E(t) - (d+\mu+\delta)I(t) \\
&\geq \left[\frac{k_1(1-\xi^*)}{k_1+\mu}\left(\beta\left(\frac{A}{\phi+\mu}-\epsilon\right) + (1-\sigma)\beta\left(\frac{\phi A}{\mu(\mu+\phi)}-\epsilon\right)\right) - (d+\mu+\delta)\right]I(t) \\
&\quad + \left[\frac{k_1(1-\xi^*)}{k_1+\mu}\left(\eta\beta\left(\frac{A}{\phi+\mu}-\epsilon\right) + (1-\sigma)\eta\beta\left(\frac{\phi A}{\mu(\mu+\phi)}-\epsilon\right)\right) + k_1\xi^*\right]E(t).
\end{aligned}$$

Let $\varsigma = \min\{\beta(\frac{A}{\phi+\mu}-\epsilon) + (1-\sigma)\beta(\frac{\phi A}{\mu(\mu+\phi)}-\epsilon) - \frac{k_1+\mu}{k_1(1-\xi^*)}(d+\mu+\delta), \frac{k_1(1-\xi^*)}{k_1+\mu}(\eta\beta(\frac{A}{\phi+\mu}-\epsilon) + (1-\sigma)\eta\beta(\frac{\phi A}{\mu(\mu+\phi)}-\epsilon)) + k_1\xi^*\} > 0$, then

$$\frac{\mathrm{d}L(t)}{\mathrm{d}t} \geq \varsigma L(t). \tag{5.1}$$

From (5.1), we can get $L(t) \to \infty$ as $t \to \infty$. However, $L(t)$ is bounded on the set $\Omega$, so the assumption above is not true.

We have proved that for each $u \in \Omega \setminus \Sigma$, with $u$ belonging to a suitably small neighborhood of $\Sigma$, there is some $T_u$ such that $P(\pi(u, T_u)) > P(u)$. Therefore, Lemma 5.1 allows us to reach to the conclusion of Theorem 5.1. This completes the proof. □

## 6 The effect of vaccination

### 6.1 Analysis of the system without vaccination

Consider the system (1.2) in the absence of vaccination (i.e., vaccination-free model), obtained by setting $V = \phi = 0$ in system (1.2), given by

$$\begin{cases}
\frac{\mathrm{d}S}{\mathrm{d}t} = A - \beta(I+\eta E)S - \mu S, \\
\frac{\mathrm{d}E}{\mathrm{d}t} = \beta(I+\eta E)S - k_1E - \mu E, \\
\frac{\mathrm{d}I}{\mathrm{d}t} = k_1E - \mathrm{d}I - \delta I - \mu I.
\end{cases} \tag{6.1}$$

It is easy to see that the region

$$\Omega_0 = \left\{(S, E, I) | S \geq 0, E \geq 0, I \geq 0, S+E+I \leq \frac{A}{\mu}\right\}$$

is a positive invariant set of system (6.1). We will consider the dynamic behavior of system (6.1) on $\Omega_0$.

The vaccination-free model (6.1) always has a disease-free equilibrium $\bar{P}_0(\frac{A}{\mu}, 0, 0)$. In the following, we will consider the locally and globally asymptotical stability of $\bar{P}_0(\frac{A}{\mu}, 0, 0)$.

Define

$$\mathcal{R}_0 = \frac{\beta A(\mu+d+\delta+k_1\eta)}{\mu(\mu+k_1)(\mu+d+\delta)}.$$

The basic reproduction number, sometimes called basic reproductive rate or basic reproductive ratio, is one of the most useful threshold parameters which characterize mathematical problems concerning infectious diseases.

**Theorem 6.1** *If $\mathcal{R}_0 < 1$, the disease-free equilibrium $\bar{P}_0$ of system (6.1) is locally asymptotically stable; if $\mathcal{R}_0 = 1$, $\bar{P}_0$ is stable; if $\mathcal{R}_0 > 1$, $\bar{P}_0$ is unstable.*

*Proof* The linearization of the model (6.1) at the equilibrium $\bar{P}_0$ gives the following characteristic equation:

$$(\lambda + \mu + \phi)\left[\lambda^2 + \left(2\mu + k_1 + d + \delta - \frac{\beta A}{\mu}\right)\lambda + \left(\mu + k_1 - \frac{\beta A}{\mu}\right)(\mu + d + \delta) - \frac{k_1 \beta \eta A}{\mu}\right] = 0. \quad (6.2)$$

It is obvious that the characteristic Eq. (6.2) has always two negative eigenvalues $\lambda_1 = -\mu - \phi$. The other eigenvalues of Eq. (6.2) are determined by the following equation

$$\lambda^2 + \left(2\mu + k_1 + d + \delta - \frac{\beta A}{\mu}\right)\lambda + \left(\mu + k_1 - \frac{\beta A}{\mu}\right)(\mu + d + \delta) - \frac{k_1 \beta \eta A}{\mu} = 0. \quad (6.3)$$

It is easy to see that all roots of Eq. (6.3) have negative real parts if and only if $(\mu + k_1 - \frac{\beta A}{\mu})(\mu + d + \delta) - \frac{k_1 \beta \eta A}{\mu} > 0$, i.e., $\mathcal{R}_0 < 1$. If $\mathcal{R}_0 = 1$, one eigenvalue of Eq. (6.3) is 0 and it is simple. If $\mathcal{R}_0 > 1$, one of roots of Eq. (6.3) has positive real parts. □

To obtain the global attractivity of the disease-free equilibrium $P_0$, we need the following lemma. Let $f_\infty = \lim_{t\to\infty} \inf_{\theta \geq t} f(\theta)$, $f^\infty = \lim_{t\to\infty} \sup_{\theta \geq t} f(\theta)$.

**Lemma 6.1** [15] *Assume that a bounded real-valued function $f : [0, \infty) \to R$ be twice differentiable with bounded second derivative. Let $k \to \infty$ and $f(t_k)$ converges to $f_\infty$ or $f^\infty$. Then $\lim_{t\to\infty} f'(t_k) = 0$.*

**Theorem 6.2** *If $\mathcal{R}_0 < 1$, the disease-free equilibrium $\bar{P}_0$ of the system (6.1) is globally asymptotically stable.*

*Proof* From the above discussion, we have obtained that the unique disease-free equilibrium $\bar{P}_0$ of the system (6.1) is locally asymptotically stable whenever $\mathcal{R}_0 < 1$. From the first equation of (6.1), we have $\frac{\mathrm{d}S(t)}{\mathrm{d}t} \leq A - \mu S(t)$. A solution of the equation $\frac{\mathrm{d}z(t)}{\mathrm{d}t} = A - \mu z(t)$ is a supper solution of $S(t)$, i.e., $z(t) \geq S(t)$ for all $t \geq 0$. Noting that $z(t) \to \frac{A}{\mu}$ as $t \to \infty$, it follows that for a given $\epsilon > 0$, there exists a $t_0$ such that $S(t) \leq z(t) \leq \frac{A}{\mu} + \epsilon$. Let $\epsilon \to 0$, we have $S^\infty \leq \frac{A}{\mu}$.

Consequently, the second equation of (6.1) can be expressed as

$$\frac{\mathrm{d}E}{\mathrm{d}t} \leq \beta(I + \eta E)\left(\frac{A}{\mu} + \epsilon\right) - (\mu + k_1)E, \quad \text{for} \quad t \geq t_0. \quad (6.4)$$

Using (6.4) and the last equation of (6.1), we have the following system:

$$\begin{pmatrix} \frac{\mathrm{d}E(t)}{\mathrm{d}t} \\ \frac{\mathrm{d}I(t)}{\mathrm{d}t} \end{pmatrix} \leq \Theta \begin{pmatrix} E \\ I \end{pmatrix} \quad (6.5)$$

with

$$\Theta = \begin{pmatrix} \frac{\beta A}{\mu} + \epsilon - \mu - k_1 & \frac{\beta A}{\mu} + \epsilon \\ k_1 & -(\mu + d + \delta) \end{pmatrix}.$$

Let $m > 0$ such that $m > \max\{\frac{\beta A}{\mu} + \epsilon - \mu - k_1, -(\mu + d + \delta)\}$. Thus, $\Theta + mJ_{2\times 2}$ is a strictly positive matrix where $J_{2\times 2}$ is the identity matrix. It is clear that if $\omega_1$ and $\omega_2$ are the eigenvalues of $\Theta$, then $\omega_1 + m$ and $\omega_2 + m$ are the eigenvalues of $\Theta + mJ_{2\times 2}$. It follows from the Perron–Frobenius theorem [18] that $\Theta + mJ_{2\times 2}$ has a simple positive eigenvalue equal to spectral radius (dominant eigenvalue) and a corresponding eigenvector $\mathbf{e} > 0$ (i.e., all components of the eigenvector $\mathbf{e}$ are positive). This implies that $\omega_1$ and $\omega_2$ are both real. If $\omega_1 + m$ is the dominant eigenvalue of $\Theta + mJ_{2\times 2}$, then $\omega_1 > \omega_2$ and $\mathbf{e}\Theta = \omega_1 \mathbf{e}$. Obviously, $\omega_1$ and $\omega_2$ are the roots of the equation

$$\lambda^2 + \left(2\mu + k_1 + d + \delta - \frac{\beta A}{\mu}\right)\lambda + \left(\mu + k_1 - \frac{\beta A}{\mu}\right)(\mu + d + \delta) - \frac{k_1 \beta \eta A}{\mu} = 0.$$

Since $\mathcal{R}_0 < 1$, for $\epsilon > 0$ sufficiently small, we have

$$\mu + k_1 - \beta\left(\frac{A}{\mu} + \epsilon\right) - k_1\beta\eta\left(\frac{A}{\mu} + \epsilon\right) > 0.$$

Hence, $\omega_1$ and $\omega_2$ are negative. It follows from (6.5) that for $t \geq t_0$,

$$\frac{\mathrm{d}}{\mathrm{d}t}(\mathbf{e} \cdot [E(t), I(t)]) \leq \omega_1 \mathbf{e} \cdot [E(t), I(t)],$$

where "·" denotes the dot product of the two vectors $\mathbf{e}$ and $[E(t), I(t))]$. Integrating the above inequality gives

$$0 \leq \mathbf{e} \cdot [E(t), I(t)] \leq \mathbf{e} \cdot [E(t), I(t)] \exp(t - t_1)$$

for $t \geq t_1 \geq t_0$. Since $\omega_1 < 0$, it follows that

$$\mathbf{e} \cdot [E(t), I(t)] \to 0 \quad \text{as} \quad t \to \infty.$$

Now, we show $\lim_{t\to\infty} S(t) = \frac{A}{\mu}$. In fact, by Lemma 6.1, we choose a sequence $t_n \to \infty$, $s_n \to \infty$ for $n \to \infty$ such that $S(s_n) \to S^{\infty}$, $S(t_n) \to S_{\infty}$, $S'(s_n) \to 0$, $S'(t_n) \to 0$. Noting that $E(t), I(t) \to 0$ for $t \to \infty$, and from the first equation of the system (6.1), we obtain

$$\begin{aligned} A\ &-\mu \lim_{t\to\infty} \sup S = 0, \\ A\ &-\mu \lim_{t\to\infty} \inf S = 0. \end{aligned} \tag{6.6}$$

It follows from (6.6) that $\lim_{t\to\infty} S(t) = \frac{A}{\mu}$.

Hence, incorporating Lemma 6.1, the disease-free equilibrium $\bar{P}_0$ is globally asymptotically stable if $\mathcal{R}_0 < 1$. □

## 6.2 Dependence on vaccination rate

We will consider the parameters $A, \eta, \beta, \mu, \sigma, k_1, \delta$ and $d$ as fixed and will view $\phi$ as variable. In practice, the parameter $\phi$ is the one most easily controlled, and later we will express our results in terms of an uncontrolled model with parameters $A, \eta, \beta, \mu, \sigma, k_1, \delta$ and $d$ fixed and examine the effect of varying $\phi$. With this interpretation in mind, we will use $\mathcal{R}_V(\phi)$ to denote the basic reproductive number of the model (1.2).

Calculating the derivative of $\mathcal{R}_V(\phi)$, we can get

$$\mathcal{R}'_V(\phi) = -\frac{\mu\beta A(\mu + d + k_1\eta)}{(\mu + k_1)(\mu + d + \delta)(\mu + \phi)^2}.$$

It is easy to see that $\mathcal{R}'_V(\phi) \leq 0$. Hence, $\mathcal{R}_V(\phi)$ is a decreasing function in $\phi \geq 0$. This indicates the impact of vaccination in reducing the vaccine-induced reproduction number. Moreover, in the absence of vaccination, i.e.,

$$\phi = V = 0, \quad \mathcal{R}_V(\phi) = \frac{\beta A(\mu + d + k_1\eta)}{\mu(\mu + k_1)(\mu + d + \delta)} = \mathcal{R}_0.$$

From the definition of $\mathcal{R}_V(\phi)$ and $\mathcal{R}_0$, it is clear that the introduction of vaccination implies $\mathcal{R}_V(\phi) \leq R_0$, and, consequently, if $\mathcal{R}_0 < 1$ then $\mathcal{R}_V(\phi) < 1$ when $w \geq 0$. Thus, $P_0$ is locally asymptotically stable as long as $\mathcal{R}_V(\phi)$ is less than one.

From (2.1), we can deduce that

$$(1 - \sigma)\mathcal{R}_0 = \frac{(1 - \sigma)\beta A(\mu + d + k_1\eta)}{\mu(\mu + k_1)(\mu + d + \delta)} \leq \mathcal{R}_V(\phi) \leq \mathcal{R}_0.$$

Thus, if $\frac{(1-\sigma)\beta A(\mu+d+k_1\eta)}{\mu(\mu+k_1)(\mu+d+\delta)} \geq 1$ then $\sigma > \sigma_* := 1 - \frac{\mu(\mu+k_1)(\mu+d+\delta)}{\beta A(\mu+d+k_1\eta)}$. This implies that $\mathcal{R}_V(\phi) > 1$ and, therefore, no amount of vaccination can bring $\mathcal{R}_V(\phi)$ below one. Hence, $\sigma_*$ defines the critical value for vaccine-related reduction rate of infection.

Also $\lim_{\phi\to\infty}\mathcal{R}_V(\phi) = \frac{(1-\sigma)\beta A(\mu+d+k_1\eta)}{\mu(\mu+k_1)(\mu+d+\delta)} := \mathcal{R}_1$, which implies that $\mathcal{R}_1 \le \mathcal{R}_0$ as $1-\sigma \le 1$. Thus, if the vaccination rate is sufficiently high then $R_1$ can be made less than one if $\sigma \to 1$. Furthermore, we can write $\mathcal{R}_V(\phi) = \frac{\mu\mathcal{R}_0+\phi\mathcal{R}_1}{\mu+\phi}$ using $\mathcal{R}_0$ and $\mathcal{R}_1$. Setting $\mathcal{R}_V(\phi) = 1$ and solving for $\phi$, we can get a threshold vaccination rate, $\phi_* = \frac{\mu(\mathcal{R}_0-1)}{1-\mathcal{R}_1}$.

Now consider $\mathcal{R}_1 < 1 < \mathcal{R}_0$, we can get $\phi_*$ positive. Here $\mathcal{R}_0 < 1$ and $\mathcal{R}_1 > 1$ are not admissible as $\varepsilon \le 1$. If $\phi > \phi_*$, then $\mathcal{R}_V(\phi) < 1$ as $\mathcal{R}_V(\phi)$ is a decreasing function for $w \ge 0$. Thus, if the vaccination coverage level $\phi$ exceeds the threshold $\phi_*$ then the disease can be eradicated provided vaccine efficacy is high enough, i.e., $\sigma = 1$.

From Eq. (1.2), we also find that,

$$\phi = \frac{(1-\sigma)\beta V^*(I^*+\eta E^*)+\mu V^*}{S^*}. \tag{6.7}$$

The critical vaccination level that ensures disease eradication, when latent population remains at its equilibrium is obtained as,

$$\phi_{**} = \frac{S^*}{\mu V^*}.$$

Therefore, if critical vaccination level $\phi$ is such that $\phi > \max\{\phi_*, \phi_{**}\}$, then disease eradication is possible in the population.

## 6.3 Implication for disease control

Since the model (1.2) has a globally stable disease-free equilibrium for $\mathcal{R}_V < 1$, it follows that the vaccination-free model (6.1) has a globally stable disease-free equilibrium for $\mathcal{R}_0 < 1$. It is instructive to determine elimination conditions in terms of the fraction $(p)$ of the population that are vaccinated at equilibrium, which is given by $p = \frac{V_0}{N_0} = \frac{\phi}{\mu+\phi}$. This enables the determination of a critical fraction that must be vaccinated at the disease-free equilibrium. The impact of the level of coverage and the "take" of the vaccine upon the vaccinated reproductive number are as one would expect [5]. From the definitions of $\mathcal{R}_V$ and $\mathcal{R}_0$, we can present $\mathcal{R}_V = \mathcal{R}_0(1-\sigma p)$. Setting $\mathcal{R}_V = 0$, and solving for the critical vaccinated fraction $p = p_c$ gives

$$p_c = \frac{1}{\sigma}\left(1-\frac{1}{\mathcal{R}_0}\right). \tag{6.8}$$

Our expression for the threshold vaccinated fraction at equilibrium, $p_c$, is the same as obtained by Hethcote [6].

From (6.8), $p_c$ is positive if $\mathcal{R}_0 > 1$, marking the case in which vaccination has a positive impact on disease control by decreasing influenza A (H1N1) prevalence. On the other hand, if $\mathcal{R}_0 < 1$, then the disease dies out without vaccination (since the disease-free equilibrium of the model (6.1) is globally asymptotically stable if $\mathcal{R}_0 < 1$). For the model (1.2) with $\mathcal{R}_0 > 1$, influenza A (H1N1) can be eliminated from the community if the fraction of individuals vaccinated at steady-state exceeds the threshold $p_c$ (i.e., $p > p_c$). The inequality $p > p_c$ can be rewritten as (Figs. 2 and 3):

$$p\sigma \ge 1-\frac{1}{\mathcal{R}_0}.$$

It is clear from (6.8) that the lower the efficacy of the vaccine (smaller $\sigma$), the higher the fraction of the population that needs to be vaccinated to attain herd immunity.

Hsieh et al. [7] have estimated that the basic reproductive number $\mathcal{R}_0$ is equal to 1.30 for the first phase (April 1 to May 4) and 1.35 for the second phase (May 4 to June 19) with the 2009 Novel A (H1N1) influenza outbreak in Canada. In this paper, we use that $\mathcal{R}_0 = 1.35$. Hence, it follows that, for an influenza A (H1N1) vaccine that offers 80% efficacy, a threshold vaccinated fraction of $p_c = 0.324074074$ must be attained in order to eliminate influenza A (H1N1) from the community. Similarly, an influenza A (H1N1) vaccine with lower efficacy, such as $\sigma = 0.259259259$, would necessitate vaccinating a much larger fraction of the population to achieve herd immunity (in this case, $p_c = 1$, requiring the vaccination of the entire population).

**Fig. 2** Variation of vaccine-induced reproduction number $\mathcal{R}_V(\phi)$ with vaccination coverage $\phi$

**Fig. 3** Contour plot of $\mathcal{R}_V$ as a function of the fraction of $\sigma$ with several values of $\mathcal{R}_0$

## 7 Numerical simulation

It is noted here that our aim is to study, through a non-linear model and its qualitative analysis, the role of vaccination on the spread of influenza A (H1N1). It is, therefore, desirable that we must show the existence of equilibrium values of variables of the system (1.2) as well as the feasibility of stability conditions numerically for a set of parameters.

To study the dynamical behavior of the system (1.2), numerical simulation of the system is done by Matlab 9.5 using the parameters in Table 1 with $\beta = 0.000018$. The equilibrium values are computed as follows: $P^*(1218.455693, 6177.887467, 72.95341274, 103.4398159)$.

The eigenvalues corresponding to variational matrix of endemic equilibrium are: $-0.3065454775$, $-0.0120$ $7189977$, $-0.009195044789 - 0.01338597026I$ and $-0.009195044789 + 0.01338597026I$.

Since all the eigenvalues corresponding to $P^*$ are negative, therefore $P^*$ is locally asymptotically stable. The computer simulations are performed for different initial starts in the following four cases and displayed graphically in Fig. 4.

(1) $S(0) = 1{,}300$, $V(0) = 8{,}000$, $E(0) = 70$, $I(0) = 110$;
(2) $S(0) = 1{,}200$, $V(0) = 7{,}500$, $E(0) = 70$, $I(0) = 100$;
(3) $S(0) = 1{,}100$, $V(0) = 7{,}000$, $E(0) = 70$, $I(0) = 90$;
(4) $S(0) = 1{,}000$, $V(0) = 6{,}500$, $E(0) = 70$, $I(0) = 80$.

The results of numerical simulation are displayed graphically in Figs. 5 and 6 with initial point $(S(0), V(0), E(0)I(0))$=(1, 200, 7, 000, 70, 100). In Fig. 5, the variation of infective population is shown for different values of vaccination efficacy $\sigma$. It is found that as vaccination efficacy ($\sigma$) increases, the infective population decreases. This signifies that only by increasing the vaccination efficacy, spread of infectious disease cannot be significantly controlled. In Fig. 6, the variation of H1N1-infected population is shown for

**Fig. 4** Variation of population in different classes with different initial data different infective rates $\beta$. It is found that as infection rate ($\beta$) decreases, the infective population decreases. This signifies that the spread of infectious disease can be controlled by decreasing the infection rate. We can decrease the infection rate through vaccination.

**Fig. 5** Variation of infective population for different vaccination efficacy

**Fig. 6** Variation of infective population for different infective rate

## 8 Discussion

In this paper, we developed and analyzed a mathematical model for influenza A (H1N1) with vaccination. We took under consideration that only a susceptible person can be vaccinated and that the vaccine is not 100% efficient. We presented the formulations of the reproductive number and the equilibria, which are the disease-free and the endemic. And we discussed the stability of disease-free equilibrium and the persistence of the system (1.2).

Stability and persistence analysis is critical in this paper since we would be able to know whether the disease-free equilibrium would be stable and the disease would persist or not. By setting up a good epidemic model and understanding well, we can have many advantages of preventing the spread of infection in the population. The most important results may be knowing the stability of the equilibria and the persistence of the system. In order to eradicate influenza A (H1N1) that is already established, we need to make sure that the vaccination reproductive number $\mathcal{R}_V$ is less than one. To obtain it, we should increase the vaccination rate. Our study shows that higher values of vaccination rate $\phi$ significantly reduce the number of infected individuals, and lead to disease eradication. Hence, our model will be useful and more accurate for humans in the following years, when we take the vaccine as the effective control measure of influenza A (H1N1). It will certainly be helpful for us to plan vaccination policy of influenza A (H1N1) better.

**Acknowledgments** We would like to thank the anonymous referees for their careful reading of the original manuscript and their many valuable comments and suggestions that greatly improved the presentation of this work. This work is supported by the National Natural Science Foundation of China (No. 11071011) and Natural Science Foundation of the Education Department of Henan Province (Nos. 2010A110017 and 2011B110028).

**Open Access** This article is distributed under the terms of the Creative Commons Attribution License which permits any use, distribution, and reproduction in any medium, provided the original author(s) and the source are credited.
